# Comparison of Biomarkers Playing a Role in Pterygium Development in Pterygium and Recurrent Pterygium Tissues

**DOI:** 10.3390/diagnostics14232619

**Published:** 2024-11-21

**Authors:** Özgür Eroğul, Serkan Şen

**Affiliations:** 1 Department of Ophthalmology, Faculty of Medicine, Afyonkarahisar Health Sciences University, 03030 Afyonkarahisar, Turkey; 2 Department of Medical Laboratory Techniques, Ataturk Vocational School of Health Services, Afyonkarahisar Health Sciences University, 03030 Afyonkarahisar, Turkey; serkan.sen@afsu.edu.tr

**Keywords:** pterygium, recurrent pterygium, IL-6, IL-8, IL-1β, TGF-β1, IL-10

## Abstract

**Background/Objectives:** Pterygium is a nonneoplastic elastotic degeneration characterized by subepithelial growth. It manifests as an ocular lesion originating from the bulbar conjunctiva, extending to the corneal surface, and reaching the visual axis in some cases. Although the exact cause is unknown, prolonged exposure to ultraviolet radiation is considered the most significant contributing factor. Chronic irritation and actinic damage are likely responsible for the typical fibrovascular reactions observed in pterygium. Additionally, growth factors, cytokines, and matrix metalloproteinases play roles in the pathogenesis of pterygium. This study compared recurrent and primary pterygium cases at the molecular level to gain new insights into the etiology of pterygium. **Methods:** Total protein was extracted from surgical samples of patients with primary and recurrent pterygium, and the levels of transforming growth factor beta 1 (*TGF-β1*), interleukin-1 beta (*IL-1β*), interleukin-6 (*IL-6*), *IL-8*, and *IL-10* were analyzed using the enzyme-linked immunosorbent assay technique. Target gene expression levels were analyzed using the ΔΔCt method after cDNA synthesis from isolated RNA, with normalization to *GAPDH* and quantification performed with SYBR Green PCR Master Mix. **Results:** Among the studied cytokines, *IL-10* levels were higher in primary pterygium than in recurrent pterygium (722.0 ± 600.9/421.4 ± 266.8) (*p* = 0.0054). Other cytokines (*IL-6*, *IL-8*, *IL-1β*, and *TGF-β1*) were detected at similar levels in both primary and recurrent pterygium (*p* = 0.2986). Additionally, the *TGF-β1* gene expression was found to be significantly upregulated in recurrent pterygium tissue compared to primary pterygium tissue (*p* = 0.034). **Conclusions:** This increase suggests that TGF-β1 may contribute to the recurrence mechanisms of pterygium through processes such as fibroblast activation and tissue remodeling. The higher levels of *IL-10* in primary pterygium compared to recurrent pterygium indicate an enhanced early protective response aimed at limiting pterygium progression and controlling the inflammatory process.

## 1. Introduction

Pterygium is a nonneoplastic elastotic degeneration characterized by subepithelial growth. It originates from the bulbar conjunctiva, extends to the corneal surface, and sometimes reaches the visual axis. This ocular surface disorder is frequently observed, especially in regions near the equator. The exact cause of pterygium remains unclear; however, some risk factors have been identified, with prolonged exposure to ultraviolet radiation being the most significant [1,2]. Pterygium is generally considered a benign and cosmetic problem. However, if left untreated, it can lead to severe visual impairment and, in severe cases, potential blindness [3]. Pterygium usually develops in the nasal region but can also occur temporally, in either direction or bilaterally. Surgery for pterygium is usually considered when conservative treatments fail to relieve symptoms or when there are concerns about visual impairment or cosmetic appearance [4,5].

Genetic factors, specifically certain genes related to DNA repair, are crucial for pterygium development. However, studies on genetic variant contributions are limited in terms of sample size and should be interpreted with caution. Actinic damage and chronic irritation are likely responsible for the typical fibrovascular reaction observed in pterygium [6]. Along with UV exposure, growth factors, cytokines, and matrix metalloproteinases are involved in the pathogenesis of pterygium, triggering proinflammatory effects. TGF-β1 has been demonstrated to stimulate fibroblasts to produce extracellular matrix proteins and cell adhesion molecules, including collagen, fibronectin, and integrins [2,4].

Pterygium may recur in some cases, depending on various factors [7]. The present study aimed to compare recurrent and primary pterygium cases at the molecular level. Therefore, the levels of transforming growth factor beta 1 (*TGF-β1*), interleukin-1 beta (*IL-1β*), *IL-6*, *IL-8*, and *IL-10* were analyzed via enzyme-linked immunosorbent assay (ELISA) after total protein extraction from surgical materials obtained from patients with primary pterygium and those with recurrent pterygium. The obtained data were used to detect differences between the two groups.

## 2. Materials and Methods

This study included individuals who presented to the ophthalmology outpatient clinic at a tertiary university hospital. Detailed patient histories were collected from these patients. Pterygium was diagnosed based on clinical features, and treatment was provided through surgical procedures. Accordingly, 45 patients (30 patients with primary pterygium and 15 patients with recurrent pterygium) were included in the study. All patients underwent sutured autograft pterygium surgery. This study was supported by the Afyonkarahisar Health Sciences University BAP Commission under project number 23.GENEL.033. All participants underwent a comprehensive ophthalmological examination, which included a slit-lamp examination, best-corrected visual acuity assessment, intraocular pressure measurement using Goldman applanation tonometry, and fundus examination. B-scan ultrasonography was also performed to evaluate orbital and ocular structures.

The inclusion criteria were as follows:

- Patients aged 18–65 years;
- Patients presenting with specific pterygium symptoms;
- Patients with primary or recurrent pterygium;
- Patients who agreed to sign the individual consent form.

The exclusion criteria were as follows:

- Failure to diagnose specific pterygium symptoms (patients with atypical pterygium, pseudopterjium);
- Patients with bleeding diathesis;
- Patients who did not provide informed consent for the study.

All cases were operated on with pterygium excision and the limbal–conjunctival transplantation technique. During pterygium surgery, adjunctive medications such as mitomycin C or 5-fluorosil were not used because they could affect the biomarkers in our study. Patients who used adjunctive medications such as mitomycin C or 5-fluorosil in their first surgeries or patients with recurrent pterygium were excluded from the study.

The surgical process of recurrent pterygiums was longer because of the intense subconjunctival fibrosis and scars from the previous surgery. Patients with pseudopterygium, which is an overgrowing membrane that is not really attached to the underlying cornea, were excluded from the study.

Topical proparacaine hydrochloride (Alcaine, Alcon) was applied to all patients 5 min before the surgery. After appropriately cleaning and covering the local area, the ocular surface was irrigated with 5% povidone–iodine and allowed to sit for 3 min. Then, povidone–iodine was removed from the ocular surface with a balanced salt solution. Lidocaine hydrochloride 20 mg/mL + epinephrine 0.0125 mg/mL (Jetocaine, Adeka) was injected under the pterygium tissue. At the limbus, the pterygium tissue was accessed with Vannas scissors and separated from the sclera. The pterygium tissue was separated from the cornea using blunt dissection, starting from the base to the apex. The pterygium residues were removed using an ophthalmic Burr device (Katena, Denville, NJ, USA), and the scleral surface was cleaned with a scalpel, extending to the cornea, limbus, and medial rectus. The pterygium was excised with scissors without reaching the caruncle. Minimal cauterization was performed as needed. In the upper temporal region, the graft area was marked with a sterile pen according to the dimensions of the conjunctival defect. Subconjunctival injection of lidocaine HCl (20 mg/mL) with epinephrine (0.0125 mg/mL) was then administered. During this procedure, care was taken to ensure the graft excluded Tenon’s tissue and included limbal tissues. The graft was then sutured to the conjunctiva with a single 8/0 polyglycolic acid monofilament absorbable suture. The area from which the graft was taken was left to heal secondarily, and the sutures were not removed. Artificial tear treatment was applied to the patients before and after the surgery.

Tissue samples removed during pterygium surgery were transported to the laboratory under sterile conditions and maintained as per specific cold chain requirements. The samples received in the laboratory were stored at −20 °C in a deep freezer until analysis.

### 2.1. Homogenization of Tissues

Due to the varying quantities of samples collected during surgery, only those weighing at least 25 mg were included in the study. Samples heavier than 25 mg were weighed and adjusted to 25 mg. Since the amount of pterygium tissue obtainable from each patient was limited, we selected samples with a minimum weight of 25 mg, as preliminary tests demonstrated that this amount yielded sufficient total protein levels for successful lysate preparation and subsequent ELISA analyses.

The weighed tissues were placed into 15 mL sterile Falcon tubes, and 1 mL of lysis buffer was added to each tube. The tissues placed in the lysis buffer were homogenized using a homogenizer (Ultra Turrax T18, Wilmington, NC, USA) at 15,000 rpm for 30 s. This process was carried out on ice and under cold chain conditions. The crude extract obtained after homogenization was centrifuged at 16,000 rpm for 15 min at 4 °C to remove nondegraded tissue and cells, and the supernatant was collected for ELISA.

The lysis buffer comprised 1% Triton X-100 (*v*/*v*), 50 mM HEPES buffer pH 7.2, 100 mM NaH_2_PO_4_·2H_2_O, and an 8% protease inhibitor cocktail (including aprotinin, PMSF, leupeptin, and NaF).

The total protein concentration of the homogenate was measured to ensure equal amounts of protein were loaded in both Western blot and ELISA kits. The total protein concentration was determined using the bicinchoninic acid (BCA) method.

### 2.2. Determination of Total Protein via Bicinchoninic Acid (BCA) Protein Assay

The BCA method was used for the colorimetric detection and quantification of total protein. This method involves reducing Cu^2+^ to Cu^1+^ in an alkaline environment, followed by highly sensitive and selective colorimetric detection using a reagent containing BCA. A purple reaction product is formed when one copper ion chelates with two BCA molecules.

The Takara BCA Protein Assay Kit was used to determine total protein levels. The study was conducted per the manufacturer’s protocol. The BCA standard solution was brought to room temperature and vortexed, whereas reagents A and B were warmed to 37 °C to prepare them for use. The reagents were then mixed in a 100:1 ratio. BCA standard solutions were prepared by diluting them with deionized water. Subsequently, 10 µL of both the BCA standard and sample were added to the wells. Two replicate measurements were performed for each concentration. Next, 200 µL of the working solution were added to the standards and samples, and the mixture was incubated at 37 °C for 30 min. The samples were read at 562 nm using an ELISA microplate reader (BioTek-Epoch, Winooski, VT, USA).

### 2.3. Determination of TGF-β1, IL-1β, IL-6, IL-8, and IL-10 Levels in Tissue Homogenates via ELISA

ELISA is a quantitative analytical method that detects antigen–antibody reactions through a color change, using an enzyme-linked conjugate and enzyme substrate. It is used to determine the presence and concentration of molecules in biological fluids.

### 2.4. Total RNA Extraction, Reverse Transcription, and Quantitative PCR

Total RNA for mRNA expression analysis was isolated using the GeneJET RNA Purification Kit (Thermo Scientific, Vilnius, LT, Catalog No: K0731). The quantity and purity of the isolated RNA samples were assessed using the Epoch Take3 plate system (Agilent, Winooski, VT, USA). Complementary DNA (cDNA) synthesis was performed following the manufacturer’s protocol with the Biorad cDNA Synthesis Kit (Cat No: BR1708891). Briefly, 1 μg of total RNA was used as a template in the PCR reaction, which was carried out using reverse transcriptase (RT). Subsequently, 1 μL of cDNA from each sample was used, and the appropriate amounts of SYBR Green PCR Master Mix, forward, and reverse primers were added according to the established protocol. The expression levels of the target genes were normalized to the housekeeping gene GAPDH. Gene expression values were then calculated using the ΔΔCt method and the equation RQ = 2(-Delta Delta C(T)) with the REST2009 Version 1 software (Qiagen, Hilden, Germany). Primer sequences and PCR conditions used in the reactions are listed in Table 1. Each assay was performed in four replicates.

### 2.5. Statistical Analysis

The normality of the data was assessed using the Kolmogorov–Smirnov test, which revealed that the data were not normally distributed in any group. Consequently, the Mann–Whitney U test was conducted to compare two independent groups with nonnormally distributed data. *p* < 0.05 was considered statistically significant.

## 3. Results

Table 2 shows the demographic characteristics of the two groups. The two groups have similar characteristics in terms of age and gender.

**Figure 1:**
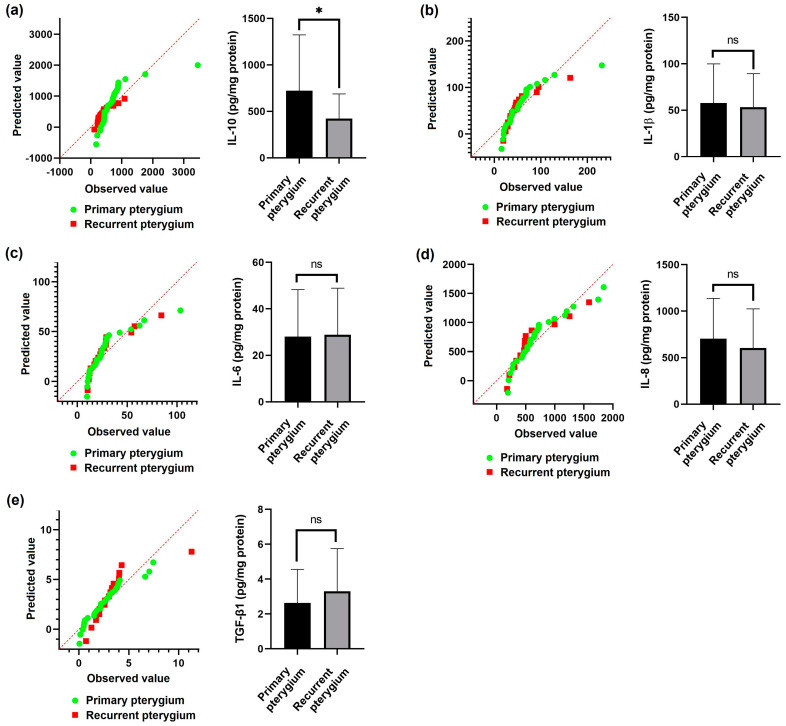
(**a**) *IL-10* QQ plot and quantification graph; (**b**) *IL-1β* QQ plot and quantification graph; (**c**) *IL-6* QQ plot and quantification graph; (**d**) *IL-8* QQ plot and quantification graph; (**e**) *TGF-β* QQ plot and quantification graph, indicating that the data are not normally distributed. “* *p* < 0.05”.

In the primary pterygium group, only *IL-10* was significantly higher compared to the recurrent pterygium group (*p* = 0.0054). The other biomarkers (*IL-6*, *IL-8*, *IL-1β*, and *TGF-β1*) gave similar results in both groups (*p* > 0.05) (Figure 1).

In Figure 1, the results of biomarkers in the primary and recurrent pterygium groups are compared.

### Comparative Analysis of mRNA Expression Levels

In our study, the mRNA expression levels of several key genes in recurrent pterygium tissue were compared with those in primary pterygium tissue. This analysis helps us better understand the biological dynamics of recurrent pterygium and sheds light on the mechanisms contributing to the recurrence of the disease.

**Figure 2:**
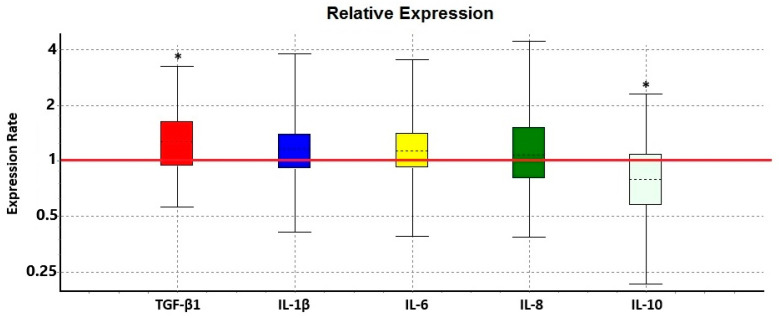
Relative mRNA expression levels of TGF-β1, IL-1β, IL-6, IL-8, and IL-10. Values are expressed as the mean ± SD. The recurrent pterygium group was compared with the primer pterygium group, and the results are given as fold increase/decrease. The REST 2009 Version 1 software (Qiagen, Hilden, Germany) was used for statistical analysis and graphing. The red line parallel to the *x*-axis shows the position of the control group. *p* < 0.05 was considered statistically significant. TGF-β1: Transforming Growth Factor Beta 1, IL-1β: interleukin 1β, IL-6: interleukin 6, IL-8: interleukin 8, IL-10: interleukin 10. “* *p* < 0.05”.

The TGF-β1 (Transforming Growth Factor Beta 1) gene showed a significant increase, with its expression being markedly upregulated in recurrent pterygium tissue compared to primary pterygium tissue (*p* = 0.034). TGF-β1 plays a pivotal role in numerous biological processes, including cell proliferation, differentiation, and tissue regeneration. Its increased expression may contribute to the recurrence of pterygium through processes such as fibroblast activation and tissue remodeling. Considering TGF-β1’s role in fibrotic diseases, these results suggest that fibrotic processes might be involved in the recurrence mechanisms of pterygium (Table 3 and Figure 2).

**Figure 3:**
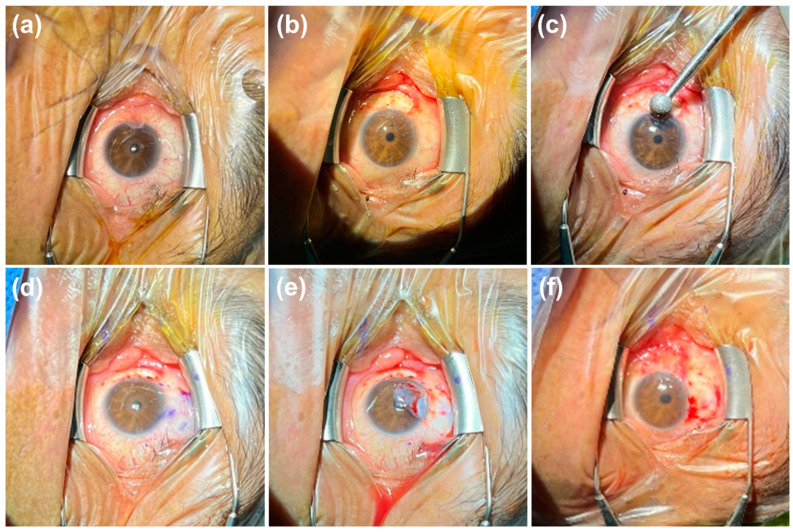
(**a**) Preoperative primary pterygium; (**b**) primary pterygium excision (bleeding is controlled with minimal cauterization); (**c**) cleaning of corneal pterygium residues; (**d**) marking the autograft site on the superior conjunctiva; (**e**) dissecting the autograft from the Tenon’s capsule; (**f**) placing the autograft in the pterygium excision area.

In Figure 3, surgical images of a patient with primary pterygium are shown. Pterygium excision was performed on patients in both groups, and the corneal area was cleaned with a Burr device after the excision. A limbal–conjunctival autograft was taken from the superior conjunctiva. Autograft suturing was performed with 8/0 polyglycolic acid monofilament absorbable sutures to secure the graft to the pterygium excision area (Figure 3).

## 4. Discussion

Pterygium is a common ocular surface disease in humans, with chronic ultraviolet (UV) exposure widely accepted as an etiological factor in its pathogenesis [5]. This hypothesis is supported by epidemiological and histopathological data related to UV-damaged skin [5,6]. Although some findings suggest that genetic factors, antiapoptotic mechanisms, and immunological mechanisms are involved in the pathogenesis of pterygium, the exact mechanism of its development is not fully understood [5,6].

Although the pathogenesis of pterygium remains unclear, epidemiological evidence suggests that environmental stress contributes to its development. Certain risk factors may play a role in the etiology of pterygium, such as UV exposure, immunoinflammatory processes, viral infections, and DNA damage [8].

In our study, there was no significant change in the mRNA expression levels of IL-1β (interleukin 1β), IL-6 (interleukin 6), and IL-8 (interleukin 8) genes (with *p* = 0.165, *p* = 0.124, and *p* = 0.324, respectively). These cytokines are generally known for their critical roles in the inflammatory response. However, the lack of significant changes in their expression in recurrent pterygium tissue suggests that the inflammatory response may not be activated through these specific genes, or that their roles in inflammation are not directly linked to pterygium recurrence.

In contrast, the IL-10 (interleukin 10) gene showed a significant decrease (*p* = 0.044). IL-10 is an anti-inflammatory cytokine known for its role in suppressing immune responses and regulating inflammation. The downregulation of IL-10 suggests that inflammatory processes may not be adequately suppressed in recurrent pterygium, pointing to the potential importance of inflammation in the recurrence of this condition. The reduced expression of IL-10 implies that the local inflammatory response may not be properly controlled, contributing to tissue recurrence.

There may be several reasons for the elevated *IL-10* levels observed in the primary pterygium group in this study. In patients with primary pterygium, a heightened anti-inflammatory response may have emerged to better regulate and control the body’s inflammation. Since *IL-10* is an anti-inflammatory cytokine, its elevated levels in this group may indicate the body’s effort to control the inflammatory response [9]. In patients developing pterygium for the first time, *IL-10* production may be increased as an early protective response to limit pterygium progression and control the inflammatory process. This may result in a more controlled and balanced inflammatory response [10]. In primary pterygium tissues, elevated *IL-10* levels may be necessary for terminating the inflammatory response and facilitating effective tissue repair processes. *IL-10* promotes tissue regeneration by regulating the functions of fibroblasts and other repair cells [11]. In patients developing pterygium for the first time, the local tissue microenvironment may establish a structure that promotes immune tolerance. In this microenvironment, increased *IL-10* production may suppress inflammation and slow the progression of pterygium [12]. In the primary pterygium group, genetic and molecular factors may contribute to elevated *IL-10* production. These factors vary depending on the individual’s genetic profiles, which regulate inflammatory responses [13].

Given *IL-10*’s role in pterygium pathophysiology as an anti-inflammatory agent, targeting *IL-10* with a specific drug could be a promising strategy for preventing recurrence. The anti-inflammatory properties of *IL-10* may suppress the inflammatory processes of pterygium and prevent its recurrence [9]. This can be achieved with a drug that either increases *IL-10* levels or mimics its effects.

Local application of such a potential therapeutic to the eye could ensure the targeted concentration of *IL-10* in the affected area, thereby minimizing systemic side effects. Alternative application methods, such as eye drops, gels, or intravitreal injections, could also be considered [10]. Additionally, *IL-10*’s tissue repair-promoting effects could support healing after pterygium surgery and reduce the risk of recurrence [11]. Furthermore, *IL-10*’s ability to induce immune tolerance may be crucial in preventing pterygium recurrence. Therefore, a drug that regulates *IL-10* levels could help balance immune responses in the pathogenesis of pterygium [12].

The similarity in *IL-6*, *IL-8*, and *IL-1β* levels between recurrent and primary pterygium groups suggests that the inflammatory response develops similarly in both groups. These cytokines are commonly produced in both primary and secondary inflammatory processes and may play a common role in pterygium pathogenesis [14,15]. Pterygium recurrence may be driven by factors other than inflammatory cytokines, such as *IL-6*, *IL-8*, and *IL-1β*. For example, growth factors and fibroblast activity may play a more decisive role in pterygium recurrence [16]. Cytokine levels can vary at different stages of the disease. Therefore, the timing of sample collection might have influenced the observed similarity in cytokine levels between the recurrence and nonrecurrence groups. In other words, samples may have been collected when the cytokine response was similar in both groups [15].

Although the pathogenesis of pterygium remains unclear, epidemiological evidence suggests that environmental stress contributes to its development. Among potential agents, UV irradiation has garnered the most attention [17]. Epithelial cells on the ocular surface produce these cytokines either as a structural component or in response to a stimulus [18].

Several functions have been proposed for *IL-6*. In most cases, ocular surface angiogenesis is detrimental to ocular tissues. Pterygium is the most common type of angiogenic activity observed in humans. One study found that *IL-6* levels increase in inflammatory events regardless of the characteristics of pterygium [19].

Besides pterygium, elevated IL-6 levels are also observed in many common pathological disorders, including corneal angiogenesis, penetrating keratoplasty, and corneal foreign bodies [20].

Levels of *IL-6*, *IL-8*, and monocyte chemoattractant protein-1 (MCP-1) are significantly elevated in the vitreous fluid in various vitreoretinal diseases, including diabetic macular edema, proliferative diabetic retinopathy, branch retinal vein occlusion, central retinal vein occlusion, and rhegmatogenous retinal detachment [21].

Elevated VEGF levels were significantly correlated with *IL-6* and *IL-8* levels. Additionally, *IL-6* and *IL-8* showed a strong correlation with each other, suggesting a common pathway involved in inflammatory and ischemic processes. IL-6 is a crucial mediator with several immune effects. Hence, completely blocking its signaling pathways may be undesirable. A clinical decision regarding *IL-6* must consider potential disadvantages, unwanted side effects, and the possibility of nonresponse in some patients [22,23].

*IL-8* is a crucial inflammatory cytokine, yet studies specifically examining its ocular effects are limited, despite its frequent mention in the literature. In the present study, we assessed the ocular effects of *IL-8* in both primary and recurrent pterygium.

In response to stimulation by Gram-positive *Staphylococcus aureus*, conjunctival epithelial cells activate the innate immune response through *IL-8* gene expression and secretion [24].

*IL-8*, along with *IL-6* and *VEGF* to some extent, is expressed by the pterygium epithelium. This expression increases with ultraviolet (UV) radiation exposure, leading to abnormal blood vessel formation, cellular proliferation, tissue invasion, and inflammation [20].

*IL-1β* is a key cytokine in ocular surface inflammation. It originates from the activation of various sources, including conjunctival epithelial cells, stromal fibroblasts, infiltrating macrophages, and lymphocytes, as well as fibroblasts from the lacrimal gland, cornea, and Tenon’s capsule [25]. Additionally, *IL-1β* is associated with the remodeling of the extracellular matrix through increased MMP activity [26].

A recent study has explored several potential targets to reduce pterygium recurrence, including *NLRP3*, *TGF-β1*, *VEGF*, *IL-6*, and *IL-8*. The researchers reported that low-dose local mitomycin C injection resulted in downregulation of IL-18 and IL-1β expression through the activation of the NLRP3/caspase-1 pathway, which in turn reduced the expression of *TGF-β1*, *VEGF*, and *IL-6.* They observed that this effectively reduced the recurrence rate of pterygium by inhibiting fibroblast proliferation and neovascularization [27].

Solomon et al. [28] suggested that environmental stimuli associated with pterygium can induce proinflammatory cytokine secretion from the ocular surface epithelium and/or inflammatory cells in the tear fluid. Among these cytokines, *IL-1β* was associated with extracellular matrix remodeling, angiogenesis, and fibroblast proliferation by activating pterygium body fibroblasts. These characteristics are important for the formation and recurrence of pterygium. They concluded that IL-1β-mediated inflammation plays a significant role in the development of both primary and recurrent pterygium.

Tsai et al. [29] found no correlation between the polymorphisms of *TNF-α* and *IL-1 TNF-α* and *IL-1β* polymorphisms and genetic susceptibility to pterygium formation and recurrence. The researchers emphasized the need for future studies to investigate other polymorphisms or haplotypes of *TNF-α* and *IL-1β* to better assess genetic susceptibility to pterygium formation and recurrence. However, no study has yet investigated *IL-1β* levels across various types of pterygium.

One study demonstrated that resveratrol effectively inhibits the synthesis of extracellular matrix proteins induced by *TGF-β1*. It also prevents myofibroblast activation, cell migration, contractile phenotype formation, and cellular proliferation by inducing apoptosis. These findings suggest that resveratrol may serve as a potential adjuvant for preventing postoperative pterygium recurrence [30].

*TGF-β* is associated with multiple aspects of pterygium pathogenesis [31,32]. Several studies have identified complex intercellular signaling networks that determine myofibroblast differentiation [32,33]. While *TGF-β*-triggered cellular transduction is a key event in many aspects of fibrotic pathogenesis, the extent to which other signaling pathways contribute to myofibroblastic differentiation remains unclear. Some studies have described a relationship between the PI3 kinase/Akt (PI3K/Akt) pathway and *α-SMA* expression during myofibroblast differentiation in various tissues [34]. Additionally, the mammalian target of the rapamycin (mTOR) pathway regulates the translation of fibronectin (FN) and the secretion and assembly of the extracellular matrix (ECM) network in pulmonary fibrosis [35].

Building on this, Kim et al. [36] found that mTOR signaling supports the profibrotic activation of human pulmonary fibroblasts (HPFs) and confirmed the importance of the mTORC2–Akt axis in *TGF-β1*-induced myofibroblast differentiation. Therefore, the researchers argued that targeting mTOR signaling could open new avenues for developing novel therapeutic strategies.

Additionally, a study reported that in atopic individuals, overexpression of the *TGF-β1* gene in pterygium tissue indicates that growth factors play a significant role in pterygium pathogenesis. The researchers noted that pterygium formation may involve fibrogenic cytokines, such as *TGF-β*, which are elevated under atopic conditions. Therefore, they recommended considering treatments that inhibit the effects of growth factors before or after pterygium tissue excision to reduce recurrence rates [37].

Due to local invasion, epithelial cell metaplasia, and abnormal expression of the *p53* tumor suppressor gene in pterygium tissue, pterygium is regarded as a benign neoplastic condition [38,39,40]. High *TGF-β1* levels suppress the immune response and promote tumor invasiveness (metastasis), cell motility, angiogenesis, and interactions between tumor cells and the extracellular matrix [41]. Therefore, elevated TGF-β1 levels are indicative of the invasiveness of various late-stage cancers [42,43].

Meanwhile, UV, smoke, and pollen are important risk factors in the etiology of pterygium. The *TGF-β1*-mediated immune response further exacerbates the pathology already demonstrated by other risk factors, such as UV light, smoke, pollen, and viruses. *TGF-β1* suppresses tumor formation. However, once tumor cells become resistant to growth inhibition, the overexpression of *TGF-β1* results in angiogenesis, invasion, and excessive extracellular matrix production.

The expression of the *TGF-β1* gene is associated with tissue fibrosis and airway remodeling [44]. Additionally, in vernal keratoconjunctivitis, *TGF-β1*, tumor necrosis factor-α, *IL-4*, and histamine release are responsible for previously described pathological changes, such as fibrillar collagen production, giant papilla formation, and conjunctival tissue remodeling [45].

Based on the current findings, *TGF-β1*, a potent mediator of tissue remodeling, induces pathological changes, including fibrovascular progression and elastotic degeneration, which are observed in pterygium.

*TGF-β1* is also crucial in wound healing. It stimulates the proliferation and migration of fibroblasts, leading to the gradual formation of a collagen matrix within the scar tissue. However, *TGF-β1*, along with insulin-like growth factor 1 and *IL-1*, can contribute to fibroproliferative disorders, such as keloids and hypertrophic scars. Inflammation, abnormalities in wound matrix remodeling, increased synthesis of extracellular matrix proteins and fibrogenic cytokines, along with cell migration, proliferation, and an enhanced response to cytokines, are believed to contribute to these conditions [46]. Photoreactive keratectomy may lead to rapid pterygium growth, possibly due to the presence of various fibrogenic cytokines during the healing period [47].

Gum et al. [48] demonstrated that cyclosporine A (CsA), which is increasingly used to treat moderate dry eye syndrome and ocular surface inflammation, inhibits the transformation of myofibroblasts induced by *TGF-β2* in primary cultured human pterygium fibroblasts. Their findings highlighted the therapeutic potential of CsA against pterygium progression.

The most important limitation of the present study was its small sample size. The sample size is generally a critical factor for such biomarker studies. A small sample size can make it challenging to detect statistically significant differences, indicating the need for larger-scale studies. Additionally, since patients with recurrent pterygium had previously undergone surgery at other centers, information about the surgical techniques used on these patients was not available.

## 5. Conclusions

Among the cytokines studied, only IL-10 levels were found to be higher in primary pterygium. Other cytokines (IL-6, IL-8, IL-1β) were detected at similar levels in primary and recurrent pterygium. These cytokines are sensitive and elevated in all inflammatory events, regardless of etiology, showing increased levels in both infection-related inflammation and noninfectious inflammatory conditions. In addition to triggering inflammation, these cytokines may also possess infection-suppressive properties. Therefore, anticytokine therapies should consider targeted approaches based on the underlying etiology. The elevated IL-10 levels observed in primary pterygium compared to recurrent pterygium may indicate an enhanced early protective response aimed at limiting pterygium progression and controlling inflammation.

The findings obtained in our study suggest that TGF-β1 and IL-10 play critical roles in recurrent pterygium, while other inflammatory cytokines do not contribute significantly, at least statistically. Given the role of TGF-β1 in fibrotic processes and the suppressive effect of IL-10 on inflammation, the changes in the expression of these genes may be crucial in the pathophysiology of recurrent pterygium. Moreover, our study provides insights into the potential for targeting these genes as a therapeutic strategy to reduce the risk of recurrence in pterygium. Future research could focus on exploring the specific roles of TGF-β1 and IL-10 in greater detail. Expanding the study to include additional cytokines and a larger participant population could offer new insights.

## Figures and Tables

**Table 1:** Oligonucleotide primer sequences and RT-PCR programs.

Genes	Primer Sequences (5′ → 3′)	RT-PCR Programs	Cycle
GAPDH	F-5′ GATTTGGTCGTATTGGGCGC 3′ R-5′AGTGATGGCATGGACTGTGG 3′	95 °C-30 s/59 °C-1 m/72 °C-30 s	35
TGF-β1	F-5′- ACGCTTCGACAATGAGACGT-3′ R-5′- CACCCTTCTCCAGCTGGAAG -3′	95 °C-30 s/57 °C-1 m/72 °C-30 s	35
IL-1β	F-5′-AACAGGCTGCTCTGGGATTC-3′ R-5′-TATCCTGTCCCTGGAGGTGG-3′	95 °C-30 s/58 °C-1 m/72 °C-30 s	35
IL-6	F-5′-CCCAGAAGTTCTCCTGCCAG 3′ R-5′-GAATCTTGCACTGGGAGGCT 3′	95 °C-30 s/57 °C-1 m/72 °C-30 s	35
IL-8	F-5′-TCTGTCTGGACCCCAAGGAA-3′ R-5′-TGGATCCTGGCTAGCAGACT-3′	95 °C-30 s/58 °C-1 m/72 °C-30 s	35
IL-10	F-5′-TACGGCGCTGTCATCGATTT-3′ R-5′-GTGGTCAGGCTTGGAATGGA-3′	95 °C-30 s/58 °C-1 m/72 °C-30 s	35

**Table 2:** Comparison of demographic characteristics between the two groups.

	Female	Male	Total Number of Patients	Average Age	*p*
Primary pterygium	19	11	30	57.60 ± 11.5	0.6808
Recurrent pterygium	10	5	15	59.13 ± 12.13	

**Table 3:** mRNA expression levels in recurrent pterygium tissue compared to primary pterygium tissue. “* *p* < 0.05”.

Genes	Recurrent Pterygium Tissues		
	Gene Expression	*p* Value	Up-/Downregulation
TGF-β1	1.253	0.034 *	Upregulation
IL-1β	1.145	0.165	Nonstatistical
IL-6	1.157	0.124	Nonstatistical
IL-8	1.118	0.324	Nonstatistical
IL-10	0.787	0.044 *	Downregulation

## Data Availability

Detailed data are available upon request from the corresponding author.
